# RETRACTED: Jakfar et al. A Polysaccharide Isolated from the Herb *Bletilla striata* Combined with Methylcellulose to Form a Hydrogel via Self-Assembly as a Wound Dressing. *Int. J. Mol. Sci.* 2022, *23*, 12019

**DOI:** 10.3390/ijms262210882

**Published:** 2025-11-10

**Authors:** Subhaini Jakfar, Tzu-Chieh Lin, Zhi-Yu Chen, I-Hsuan Yang, Basri A. Gani, Diana Setya Ningsih, Hendra Kusuma, Chia-Tien Chang, Feng-Huei Lin

**Affiliations:** 1 Institute of Biomedical Engineering, National Taiwan University, Taipei 106, Taiwan; subhaini@unsyiah.ac.id (S.J.); jacklin7412@gmail.com (T.-C.L.); d79010340217@gmail.com (Z.-Y.C.); tony910028@gmail.com (I.-H.Y.); 2 Dentistry Faculty, Syiah Kuala University, Darussalam, Banda Aceh 23111, Indonesia; basriunoe@unsyiah.ac.id (B.A.G.); dee_aceh@yahoo.com (D.S.N.); 3 Agriculture Faculty, Gajah Putih University, Aceh Tengah 24552, Indonesia; hendra.jakfar@yahoo.co.id; 4 Institute of Biomedical Engineering and Nanomedicine, National Health Research Institutes, Miaoli 350, Taiwan; aaiaaiaai0813@gmail.com

The journal retracts the article titled “A Polysaccharide Isolated from the Herb *Bletilla striata* Combined with Methylcellulose to Form a Hydrogel via Self-Assembly as a Wound Dressing” [1], cited above.

Following the publication, concerns were brought to the attention of the Editorial Office regarding the scientific accuracy of the data presented in the published paper [1].

Adhering to our complaints procedure, the Editorial Office and Editorial Board conducted an investigation that confirmed inconsistencies within the data presented in Figures 2c,d and 7a, and Table 2 [1]. Following discussions, the authors were unable to satisfactorily explain the above-mentioned concerns, nor could they provide appropriate raw material for Editorial Board evaluation.

Consequently, the Editorial Board has lost confidence in the reliability of the findings and decided to retract this publication [1], as per MDPI’s retraction policy (https://www.mdpi.com/ethics#_bookmark30).

This retraction was approved by the Editor-in-Chief of the *International Journal of Molecular Sciences.*

The authors did not agree to this retraction.
